# Predictive Modeling for Diagnostic Tests with High Specificity, but Low Sensitivity: A Study of the Glycerol Test in Patients with Suspected Menière’s Disease

**DOI:** 10.1371/journal.pone.0079315

**Published:** 2013-11-18

**Authors:** Bernd Lütkenhöner, Türker Basel

**Affiliations:** ENT Clinic, Münster University Hospital, Münster, Germany; Cleveland Clinic Lerner Research Institute, United States of America

## Abstract

A high specificity does not ensure that the expected benefit of a diagnostic test outweighs its cost. Problems arise, in particular, when the investigation is expensive, the prevalence of a positive test result is relatively small for the candidate patients, and the sensitivity of the test is low so that the information provided by a negative result is virtually negligible. The consequence may be that a potentially useful test does not gain broader acceptance. Here we show how predictive modeling can help to identify patients for whom the ratio of expected benefit and cost reaches an acceptable level so that testing these patients is reasonable even though testing all patients might be considered wasteful. Our application example is based on a retrospective study of the glycerol test, which is used to corroborate a suspected diagnosis of Menière’s disease. Using the pretest hearing thresholds at up to 10 frequencies, predictions were made by K-nearest neighbor classification or logistic regression. Both methods estimate, based on results from previous patients, the posterior probability that performing the considered test in a new patient will have a positive outcome. The quality of the prediction was evaluated using leave-one-out cross-validation, making various assumptions about the costs and benefits of testing. With reference to all 356 cases, the probability of a positive test result was almost 0.4. For subpopulations selected by K-nearest neighbor classification, which was clearly superior to logistic regression, this probability could be increased up to about 0.6. Thus, the odds of a positive test result were more than doubled.

## Introduction

An ideal diagnostic test has a high sensitivity combined with a high specificity. However, tests that are used in daily routine do rarely conform to this ideal. Instead, it is often necessary to find a reasonable trade-off between sensitivity and specificity. This means that a high sensitivity is achieved by accepting a relatively low specificity and, conversely, a high specificity is reached by compromising sensitivity. Specific tests are typically used to confirm (or “rule in”) a suspected diagnosis [1]. Specificity is synonymous for true-negative rate, 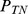, which is related to the false-positive rate, 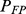, as 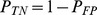. A high specificity implicates that it is unlikely to get a positive result in a patient that does not have the disease tested for. Thus, a positive outcome of a specific test is quite informative and can have substantial therapeutic consequences. A negative result, by contrast, is of little value if the sensitivity of the test is low. This follows from the fact that sensitivity is synonymous for true-positive rate, 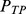, which is related to the false-negative rate, 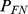, as 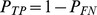. Because a low sensitivity implicates a high false-negative rate, a negative outcome of an insensitive test is a likely event that does not give a compelling reason to rule out the disease tested for.

The performance of a diagnostic test can be illustrated by making use of the so-called ROC (receiver operating characteristic) space, which is a two-dimensional scheme where the horizontal axis represents the false-positive rate (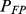, identical with 1 minus specificity) and the vertical axis represents the true-positive rate (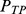, corresponding to the sensitivity). A binary classification (e.g., disease present or absent) is represented by a single point in ROC space. A diagnostic test resulting in a continuous variable can be reduced to a binary classification by specifying a threshold (cut-off) [2]. Variation of this threshold affects the trade-off between sensitivity and specificity so that the ROC space representation of the test is no longer a single point, but a curve: the ROC curve[3]–[9]. The ROC space is ideally suited to compare the performances of alternative tests in terms of sensitivity and specificity. However, these two measures alone are insufficient to assess how useful a given test is in clinical practice and how its utility compares to other tests. The reason is that a ROC space representation is independent of class probabilities (i.e., the prevalences of disease and “non-disease”) and that a ROC analysis does not account for misclassification costs (i.e., the costs for false-positive and false-negative test results) [10]. However, both aspects are of crucial importance for a cost-benefit analysis of a test.

A positive outcome of a specific test corroborates the hypothesis that the investigated patient has a certain condition (e.g., suffering from a suspected disease, possibly restricted to a certain stage). If the sensitivity is low or moderate, the test is most useful when it is applied to a population of patients that were pre-selected based on the results of previous, more unspecific tests. For the sake of simplicity, we refer to this population as the population of candidate patients (the patients may, for example, be suspected of having a certain disease). The term ‘test’ is understood here in a broad sense, which in the definition by Power et al. [11] *“includes not only laboratory tests, imaging investigations and diagnostic procedures, but also questions in history-taking and items in the physical examination"*. We extend this definition by also including approaches which reach a diagnostic conclusion from the combination of various clinical data by means of a mathematical model.

For many specific tests there is little doubt that each candidate patient should be tested. This is especially clear if the test causes no harm or major discomfort to the patient, the cost is low, and the benefit is obvious and uncontroversial. It is also clear that a practice which provides minimal or no health benefit is typically wasteful, regardless of its cost [12]. A more detailed cost-benefit analysis is mainly of interest for cases in between, where it is not so easy to decide whether the benefit outweighs the cost. As the exact measures of costs and benefits are often debatable [13], different investigators may come to different conclusions about the cost-benefit ratio. Thus, tests falling into the latter category may be controversial and not generally accepted.

To understand why a cost-benefit analysis decisively depends on the prevalence of the condition tested for, it is useful to make the simplifying assumption that, in the case of a specific test with relatively low sensitivity, a patient benefits only from a positive outcome. The expectation of the benefit is then proportional to the prevalence. As a consequence, the ratio of expected benefit and cost can be less than unity if the prevalence of the condition tested for is too low in the population of candidate patients. Under such circumstances one would generally decide against the test (although cost-effectiveness is not always crucial for providing medical interventions [12]). In deciding so, it is implicitly assumed that the probability of the condition tested for is the same for all candidate patients. However, this presumption is not necessarily true. Although all candidate patients fulfill, by definition, certain criteria, they may considerably differ with respect to parameters that are not covered by the criteria. Even parameters covered may differ. For example, if a criterion for including a patient is that a certain parameter exceeds a specific threshold, some patients may fulfill this condition much more clearly than others.

The question addressed in this article is to what extent differences within the population of candidate patients can be utilized to estimate, individually for each patient, a pre-test probability that predicts the outcome of the considered test better than does the prevalence calculated on the basis of *all* candidate patients. Such predictive modeling could be used to identify candidate patients for whom the ratio of expected benefit and cost reaches an acceptable level so that testing is reasonable even though testing the whole population might be considered wasteful. Thus, in essence, the idea is to use predictive modeling for defining a subpopulation of candidate patients in which the prevalence of the condition tested for is higher than in the total population. The motivation for this study came from our recent retrospective analysis of a special test: the glycerol test, which is used to corroborate a suspected diagnosis of Menière’s disease [14]. But the methodological approach presented below is quite general, and most concepts are almost universally applicable. With this in view, the main ideas will be presented in a way that they can be applied to basically any diagnostic test. A detailed analysis of our glycerol-test data then serves as an illustrative example.

Some of the key concepts presented below are typically applied in a different context, which may give rise to confusion. Thus, a few clarifications shall be made in advance in order to prevent misunderstanding. In essence, we propose a two-stage design, where a laborious gold standard test is reserved to patients who had a positive result in a preceding screening test. Indeed, referring to the broad-sense definition given above, predictive modeling with the aim to estimate the prospects of a considered diagnostic test may itself be regarded as a diagnostic test, which is virtual in nature, though: Instead of requiring a renewed interaction with the patient, the procedure relies on information from previous tests that was not fully exploited when assigning the patients to the population of candidates. This means that decision making based on predictive modeling may be regarded as screening. The glycerol test, on the other hand, can formally be considered as a gold standard test, although not for Menière’s disease per se, but for a specific stage of the disease (not necessarily implying that all patients pass through that stage). The evaluation of data gathered in such a two-stage design is complicated by the fact that patients screened negative are not further investigated (see, e.g., section 7.2 in [15]). But this is an issue only for future investigations. The present study is not affected by this problem, because it relies on data that were obtained by unconditionally testing all candidate patients. As to the ROC analyses presented below, the goal is to assess how well specific prediction methods (“virtual screening tests”) are able to forecast the outcome of a medical test (glycerol test in our example). By contrast, comparable analyses in other articles typically aim to assess how well a medical test can detect a disease. Hence, when comparing methodological aspects it should be born in mind that, with respect to the theoretical framework, “result of predictive modeling” and “test result” may correspond to “test result” and “disease status” in other studies where the context is different. Finally, we want to emphasize that this study is not about evaluating given methods. The original objective was to find a reasonable prediction method for the problem at hand (considering the limited amount of data available, we refrained from seeking for an “optimal” method) and to answer the question under what assumptions a prediction is useful at all.

## Methodological Approach

### Predictive Modeling: Inference and Decision

The data relevant for predicting the outcome of a considered diagnostic test are assumed to be organized as a vector of 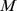 predictors, 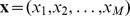. The predictors may be imagined as the results of previous tests, where the term ‘test’ refers to the broad-sense definition given in the Introduction. The basis for the prediction is provided by data from 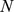 previous patients (training set). It is assumed that for each of these patients not only the vector of predictors, 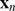, is available, but also the outcome of the test, 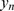 (the index 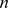, 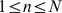, represents a consecutive patient number). This study is confined to binary classifications, where the values 0 and 1 correspond to a negative and a positive outcome, respectively. The corresponding classes are referred to as 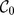 and 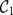. What exactly “positive outcome” means is often a matter of definition. In fact, a test may first result in a continuous variable so that it is necessary to reduce this outcome, in a subsequent step, to a binary classification, for example by requiring that a certain threshold is exceeded.

The objective of predictive modeling is to forecast, on the basis of the data from the 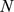 previous patients, the test result (class membership) for a new patient, for whom the vector of predictors 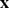 was obtained. Two probabilistic approaches are used for that purpose. The first approach, K-nearest neighbor classification, goes back to ideas developed by Fix and Hodges in 1951, which were only published many years later [16], [17]. The applicability of the method is limited by the fact that it cannot readily be used with predictors corresponding to categorical variables (although there are ways to overcome this restriction [18]). If the vectors of predictors of both the previous patients and the new patient are represented as points in an 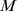-dimensional Euclidean space (possibly after an appropriate metric adjustment [19]), the class membership of the new patient can be predicted by determining the nearest neighbours of this patient’s vector of predictors, 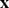, looking up the respective class labels, and finally taking a majority vote [20]. Alternatively, instead of deciding straight away, a posterior probability of class membership can be calculated as 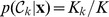, where 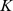 is the number of neighbours considered and 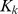 is the number of neighbours from class 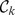 [21]. The latter approach has the advantage that it is, for example, possible to account for the costs of false decisions, as will be explained below.

The second method is logistic regression, which represents an application of the generalized linear model[22]–[24]. In essence, the posterior probability of class 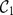 is obtained by evaluating the logistic sigmoid function,
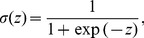
(1)for an argument corresponding to a linear combination of the predictors [21], i.e.,



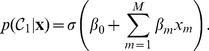
(2)The parameters 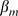 (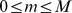) are estimated by maximum likelihood, i.e., they are determined such that the probability of obtaining the observed data is maximized [25]. In the present study this was accomplished by calling the function “ glmfit” (with the link function “ logit”) of the MATLAB Statistics Toolbox (The Mathworks, Inc., Natick, MA).

What the two methods have in common is that they provide posterior probabilities of class membership. In a subsequent step, these posterior probabilities can be used to decide whether or not the new patient should be tested. The decision has to take into account the cost for performing the test as well as possible harms and benefits. This can be formalized by introducing a loss matrix 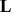 (or, alternatively, a utility matrix defined as 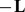): The loss that results from assigning a patient with a true class 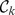 to class 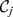 corresponds to the element 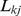 of the loss matrix (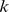 and 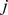 may be identical) [21]. The optimal decision is the one that minimizes the expected loss. The corresponding decision rule is to assign a new patient with predictor 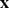 to class 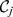 if and only if the expected loss for this decision,
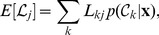
(3)is smaller than the expected loss for any other decision [21]. With only two classes, and because of 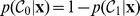, the rule for an assignment to class 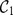 becomes 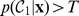, where 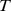 is a threshold probability defined as
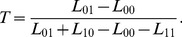
(4)With 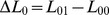 and 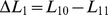 the equation can be rewritten as



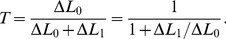
(5)The same formula was obtained by Pauker and Kassirer [26], although in a somewhat different context (therapeutic decision making). They denoted 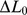 as the cost and 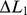 as the benefit. Our understanding of cost and benefit is somewhat different. Conceptually, we define ‘cost’ as the loss that would be caused by performing a diagnostic test and accidentally erasing the result before anybody could take notice of it. The difference in loss between testing and not testing a patient is then the cost for testing minus the benefit of the information provided by the test. If 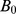 and 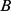 (with 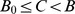) are the benefits in the case of a negative and a positive outcome, respectively, we get 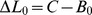 and 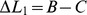, and (5) simplifies to
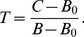
(6)


Provided that the sensitivity of the considered diagnostic test is relatively low, as supposed in this study, it is justified to make the simplifying assumption that a negative outcome has no benefit, i.e., 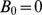. The threshold 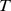 then corresponds to the cost-benefit ratio 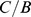. A small benefit 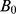 does not invalidate this interpretation: 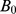 reduces both the cost 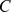 and the benefit 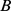 so that 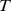 may be interpreted as the ratio of the effective cost and the effective benefit.

### Evaluation of Predictive Modeling by Cross-validation

At least as important as knowing how to predict the outcome of a considered diagnostic test is being able to evaluate the quality of the prediction, which is the basic prerequisite for comparing the performances of different approaches and their numerous variants. The basis for the prediction are the data from 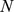 previous patients, and exactly these data can be used to evaluate an algorithm before applying it to make predictions for new patients. The method which makes this possible is cross-validation. In the leave-one-out approach [21], [27], [28] that will be used here, each of the previous patients serves, in turn, for validation while the other 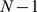 patients represent the respective training set.

Cross-validation provides an easy way not only to compare different approaches such as K-nearest neighbour classification or logistic regression, but also to investigate which combination of predictors yields the best prediction. Although this is an attractive possibility, there is also a risk, because the procedure violates the requirement that *“when doing comparative evaluations, everything that is done to modify or prepare the algorithms must be done in advance of seeing the test data”* [29]. Thus, if cross-validation is used to sift the approach with the best performance out of a greater number of alternatives, the capability to make correct predictions for unseen data (known as generalization) may be low for the “winning” approach, and this problem worsens as the number of cases in the training set decreases [30]. To be able to recognize this problem, which is due to overfitting, it is necessary to keep aside a certain proportion of the dataset (test set) until these data are eventually used for assessing the performance of the finally selected approach [21]. A successful prediction can provide evidence that overfitting has not occurred [31].

### ROC Curve, Likelihood Ratio, and Related Issues

The performances of different approaches can be compared by considering ROC curves. This necessitates to count, for a given threshold 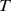, the number of true-positives, 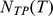, false-positives, 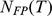, true-negatives, 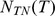, and false-negatives, 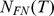. The false-positive rate (i.e., 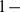 specificity), is then calculated as
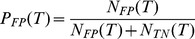
(7)and the true-positive rate (sensitivity) as



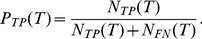
(8)Moreover, the rate of positive predictions (corresponding to the proportion of candidate patients that would be selected for testing by the predictive model) can be calculated as
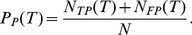
(9)


Of interest are also the positive and the negative predictive value [2], [9]. In the present context, the positive predictive value,
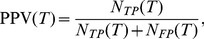
(10)is the probability that a positive prediction as to the outcome of a diagnostic would be confirmed by actually doing the test. Accordingly, the negative predictive value,
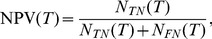
(11)is the probability that a negative prediction would be confirmed.

A plot of 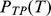 versus 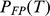 yields the ROC curve, as described in the Introduction. If different ROC curves are to be compared it is convenient to characterize each curve by a single number. The most popular one-number summary index is the area under the curve (AUC) [2]. In this study, ROC curves were obtained using the function “ perfcurve” of the MATLAB Statistics Toolbox, which readily provides the AUC. If a binary classification is based on a normally distributed continuous variable which, for class 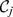, has a mean of 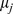 and a standard deviation of 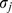, the AUC is
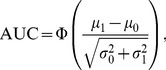
(12)where 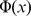 is the cumulative distribution function of a standard normal distribution [2], [32]. With the definition



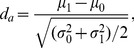
(13)Eq. (12) can be rewritten as 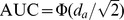, and solving for 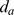 yields
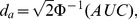
(14)where 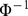 is the inverse of function 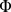. The measure 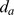, called area related index [33], provides an alternative way to characterize a ROC curve. The advantage of this measure is that it can be interpreted as a signal-to-noise ratio. In fact, under the assumption of equal variances (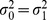), which appears to be reasonable for the data considered in this article, 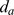 reduces to the widely used index of dectability 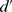 [33], [34]. The ROC curve corresponding to that simple model has the parametric representation 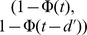.

The ratio of true-positive and false-positive rate is known as the *likelihood ratio positive*,
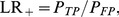
(15)which can be used to transform a pre-test probability (corresponding to the prevalence of the condition tested for) into a post-test probability [35], [36]. For such a transformation it is convenient to work with odds instead of probabilities. The odds corresponding to a probability 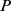 are 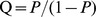, and given odds can be converted into the corresponding probability using the formula 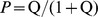. If 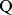 denotes the pre-test odds, then the post-test odds are simply 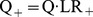.

### Threshold Dependence of the Loss

After having investigated 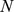 patients, the expected loss for testing a single patient can be estimated as
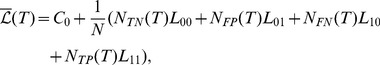
(16)where 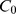 denotes the cost caused by the prediction itself. If not explicitly stated otherwise, the prediction is assumed to be made at minimal expense on the basis of readily available data so that 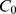 will be set zero, for the sake of simplicity. In what follows, 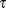 is the probability that a randomly selected candidate patient is tested positive (prevalence of a positive test result). The above equation can then be rewritten as
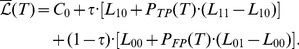
(17)This reformulation (which is equivalent to Eq. (3.5) in [37] for 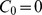 and to Eq. (2.17) in [15] for 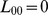, although the contexts differ) makes clear that not the individual losses, but only the differences 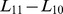 and 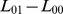 are relevant when it comes to comparing two prediction methods or the same method at different thresholds (the terms 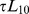 and 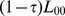 cancel out if two expected losses are subtracted). Thus, two of the four losses can be chosen ad libitum. Here, this indeterminacy is resolved by postulating that a perfect classifier has a loss of zero, which requires 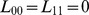. Referring to the above considerations about costs and benefits we therefore get
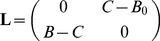
(18)as the loss matrix. Equation (16) then simplifies to
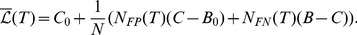
(19)A false-positive classification contributes the effective cost, 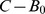, whereas a false-negative classification contributes the missed effective benefit, 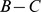. If the predictive model works well, minimization of (19) with respect to 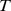 should yield a value that is roughly consistent with the threshold obtained from (6).

In other contexts it can be advantageous to use a different loss matrix. Here, we also consider the case that, in a research project, a certain diagnostic test is an integral component of an elaborate investigation which has to be completed before the outcome of the test finally becomes available. But the investigation is assumed to be of minor value if the outcome of the test is negative. It is assumed, furthermore, that an ethical review board gave the permission to investigate up to 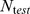 subjects. The goal of predictive modeling, then, is to ensure that as many of the 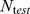 subjects as possible are tested positive, while it may be acceptable to falsely reject a large proportion of candidate subjects as being ineligible for the test (false negatives). Under such circumstances, 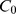 comprises not only the cost for making the prediction, but also the costs for finding candidates and doing preinvestigations. These screening costs may be quite substantial, setting an upper limit for the number of subjects that can be screened. A suitable loss matrix is
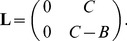
(20)


The cost 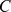 is to be paid for each patient tested (true-positive as well as false-positive predictions), whereas a benefit 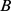 is received only for true-positive predictions.

As in the consideration before, the threshold 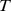 has to be determined such that the expected loss is minimized. However, Eq. (16) is not applicable here, because it is based on the assumption that the total number of subjects, 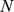, is a known constant. But the number of subjects screened depends on 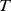 now. To get a formula for the expected loss, it is helpful to first ask the question as to how often the three losses (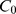, 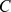, and 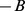) can be expected to arise. The number of patients required for screening (each causing the loss 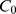) can be estimated by dividing 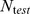 by the rate of positive predictions, 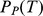. The number of subjects actually tested (each causing the cost 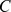) is, by definition, 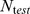. The number of tested subjects for which we can expect a positive outcome (providing the benefit 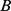) is obtained by multiplying 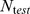 by the positive predictive value, 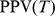. In summary, the expected loss for testing one of the 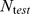 subjects is
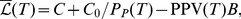
(21)where 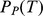 and 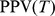 are calculated using Eqs. (9) and (10), respectively.

## An Illustrative Example: The Glycerol Test

### Background

Menière’s disease is characterized by episodes of vertigo, hearing loss, and tinnitus or aural fullness. As yet, the diagnosis is mainly based on the case history, the exclusion of other diseases, and an elementary investigation of the patient’s hearing abilities. Many efforts have been made to find objective correlates of Menière’s disease [38], [39], but there is still no method that could be considered as the ‘gold standard’. One of the methods proposed is the glycerol test [40], which relies on the fact that, in Menière patients, oral application of glycerol can temporarily improve the threshold of hearing, whereas the threshold does not systematically change in normal hearing subjects or patients with other hearing disorders. The glycerol test perfectly fits the category of tests that was the focus of the above methodological considerations: Although the test is assumed to have a high specificity, it is discussed controversially because it is time-consuming, somewhat uncomfortable for the patient, and not overly sensitive.

The glycerol test involves a comparison of two pure-tone audiograms: The first one (pretest audiogram) is obtained immediately before glycerol is orally administered to the patient, and the second one is obtained a couple of hours later (four hours in this study). The outcome is considered positive if the second audiogram provides evidence of improved hearing (lower thresholds in a certain frequency range). Many suggestions have been made as to how the improvement should be ascertained. The criterion used here is an aggregate threshold reduction (ATR) of at least 30 dB in a contiguous frequency range. The criterion is associated with a false-positive rate of approximately 5% [14].

### A First Look at the Data

In a previous article [14] we presented a retrospective analysis of 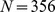 glycerol tests in patients with suspected Menière’s disease. Among other things, it was shown that the probability of a positive test result depends on the pretest audiogram. A typical audiogram comprises the thresholds of hearing at about 10 distinct frequencies. While the thresholds are normally displayed in a standardized form as hearing loss versus frequency, they may also be arranged as a vector of predictors, 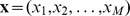. It can be useful to reduce the dimensionality of the original data, for example by ignoring some frequencies or averaging thresholds over certain frequency ranges. Moreover, it may be advantageous to transform the original data, for example by subtracting two thresholds. As a consequence, the number of predictors, 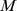, may be smaller than the number of estimated thresholds.

**Figure 1:**
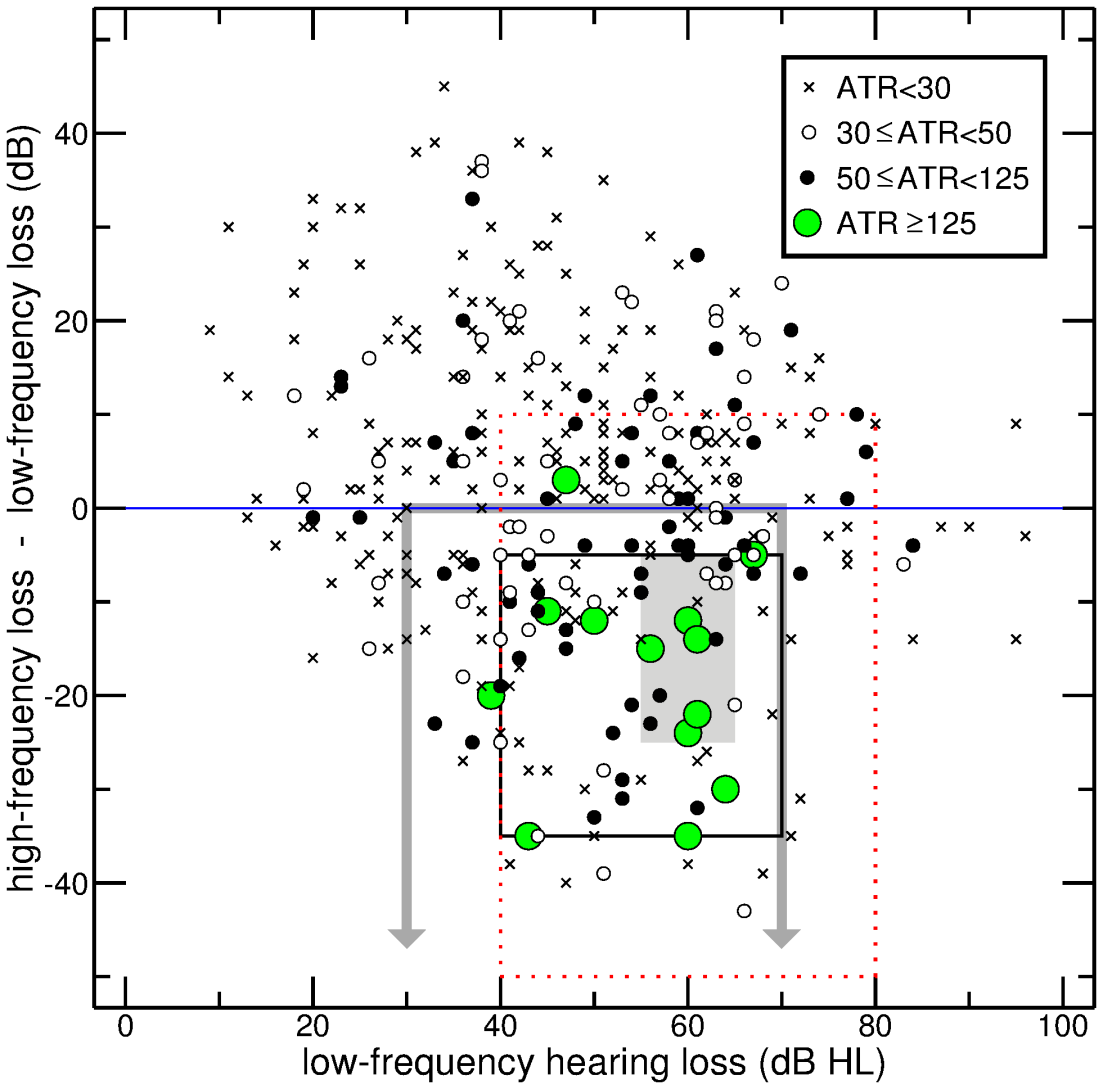
The results of all 356 glycerol tests projected into a two-dimensional predictor space. The two axes represent the mean low-frequency hearing loss (horizontal) and the difference between mean high-frequency and mean low-frequency hearing loss (vertical). The type of symbol indicates the aggregate threshold reduction (ATR) that was observed four hours after the glycerol intake. The gray area indicates the optimal region under the constraint that at least 5% of the cases are to be included. Optimal regions with at least 20% and 50% of the cases are bounded by solid and dotted lines, respectively. The area framed by thick gray lines with downward arrows represents a simple rule of thumb[?] according to which a glycerol test should be considered as a diagnostic option when a patient with suspected Menière’s disease has a mean low-frequency hearing loss between 30 and 70 dB and a mean high-frequency hearing loss that does not exceed the mean low-frequency loss.

To illustrate the predictive value of the pretest audiogram, Fig. 1 shows all test results as points in a two-dimensional predictor space: the abscissa represents the mean low-frequency hearing loss (frequency range 150 to 1500 Hz), whereas the ordinate represents the difference of mean high-frequency hearing loss (frequency range 2000 to 8000 Hz) and mean low-frequency hearing loss. The outcome of the test is indicated by the type of symbol. Most common is a small cross, which indicates a negative outcome (ATR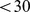). Circles, by contrast, represent a positive outcome (the two types of filled circles indicate strong and very strong effects, repectively). The distribution of the crosses clearly differs from the distribution of the circles, especially from the distribution of the big circles, which represent cases with ATR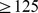: Symbols representing patients that were tested positive are more likely to be found below the zero line, which means that the hearing loss is more pronounced in the low-frequency range. A related observation in our previous article [14] gave rise to a simple rule of thumb: A positive outcome of the glycerol test is particularly likely if the mean low-frequency hearing loss is between 30 and 70 dB and the mean high-frequency loss does not exceed the mean low-frequency loss. In Fig. 1, this concept is visualized as an area framed by thick gray lines (the arrows indicate that there is no lower bound).

To draw quantitative conclusions, an exhaustive search algorithm was used to identify regions where the probability of finding an ATR of at least 30 dB is as high as possible (the borders were systematically changed in steps of 5 dB; a similar analysis was described in our previous article [14]). In Fig. 1, the area shaded in gray is the optimal region under the constraint that at least 5% of the cases are to be included. Solid and dotted lines bound optimal regions comprising at least 20 and 50% of the cases, respectively. More information is provided in Table 1, where also two additional constraints (inclusion of at least 10% and 100% of the cases, respectively) are considered. In the upper half of the table, the optimized regions are defined, and it is stated how many cases are actually found in those region. The lower half of the table provides the probabilities that specific criteria are fulfilled. The rightmost column was not obtained by optimization, but represents the simple rule of thumb explained above, for the sake of comparison. The table shows that the probability of obtaining a positive test result can be substantially increased by testing only a subpopulation of patients. For new patients, somewhat lower probabilities are to be expected, though (see Discussion).

**Table 1:** Optimal pretest conditions for the criterion ATR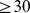.

	Percentage of cases to be included					Rule of thumb
	≥5	≥10	≥20	≥50	100	
Actual number of cases	21	36	76	182	356	127
Pretest loss (dB HL)						
at low frequencies	60±5	55±10	55±15	60±20	50±50	50±20
high minus low freqs.	−15±10	−15±10	−20±15	−20±30	0±45	≤0
Probability						
of ATR ≥30	0.86	0.75	0.67	0.51	0.39	0.57
of ATR ≥50	0.62	0.56	0.46	0.31	0.22	0.37
of ATR ≥125	0.24	0.19	0.14	0.066	0.037	0.094

### Predictive Modeling

A two-dimensional predictor space such as the one considered in Fig. 1 has the advantage of being well-suited for visualization. However, important information might get lost by reducing the dimensionality of the data. In fact, the starting point for this study was the question to what extent more sophisticated methods of predictive modeling are superior to the above-mentioned rule of thumb.

**Figure 2:**
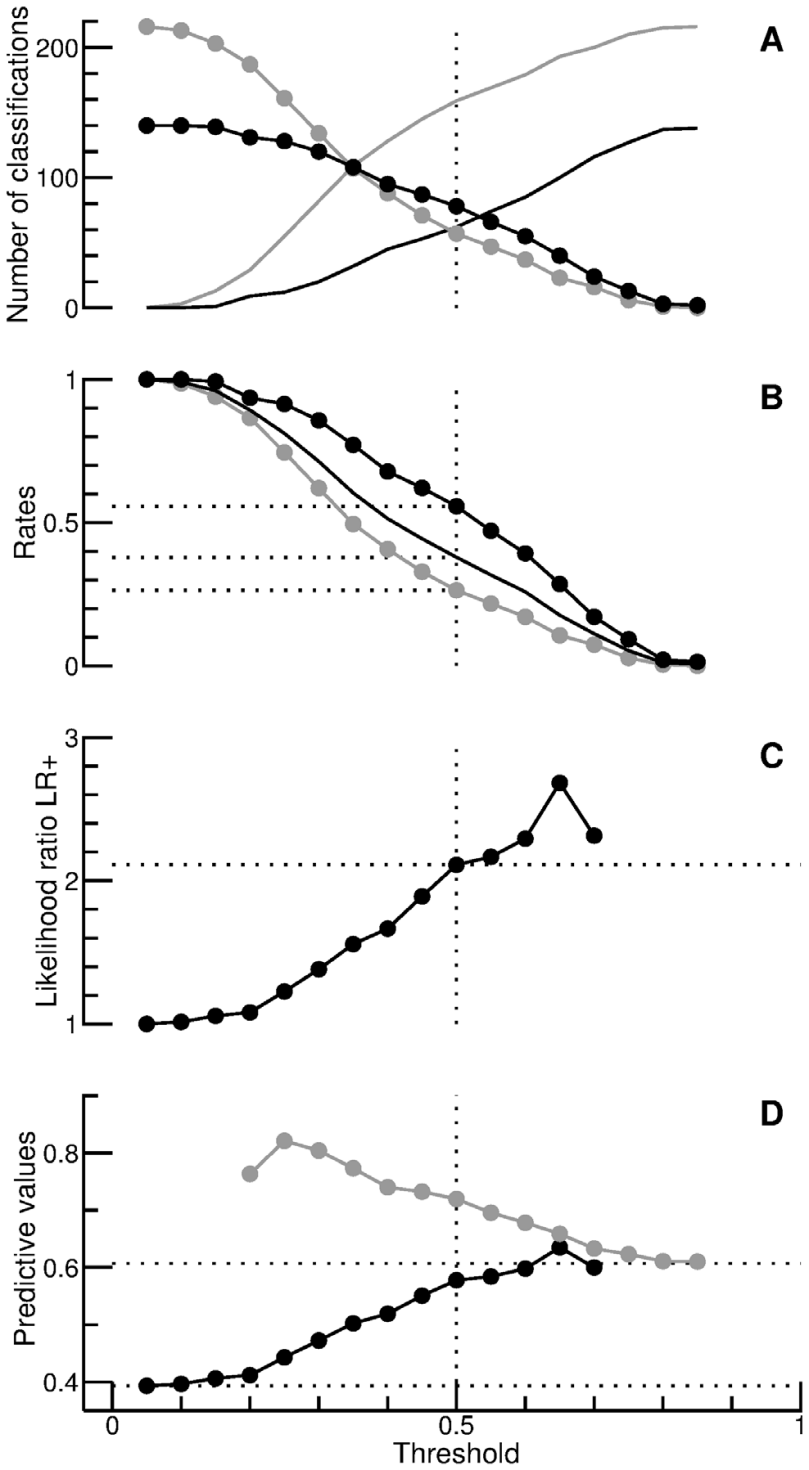
Prediction of a positive outcome of the glycerol test by K-nearest neighbor classification. (A) The curves with filled circles show, as a function of threshold, the numbers of true-positive (black) and false-positive predictions (gray). The other curves show the numbers of false-negative (black) and true-negative predictions (gray). (B) The curves with filled circles show the true-positive rate (black) and the false-positive rate (gray). The other curve shows the rate of positive predictions, corresponding to the proportion of candidate patients that would be subjected to testing. (C) Likelihood ratio 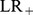. (D) Positive predictive value (black) and negative predictive value (gray).

An evaluation of the K-nearest neighbor approach (with 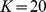) by means of leave-one-out cross-validation is provided in Fig. 2. The pre-test hearing losses at seven distinct frequencies (125–3000 Hz) served to predict the outcome of the glycerol test, and this prediction was checked against the true outcome. The choice of the frequency range reflects the fact that higher frequencies are generally of minor importance for Menière’s disease. Panel A shows, as a function of threshold 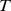, the number of true-positive predictions (black curve with filled circles) and false-positive predictions (gray curve with filled circles). The complementary curves, representing the number of false-negative (black) and true-negative predictions (gray), are shown as well, for the sake of completeness. An appropriate rescaling yields the true-positive and false-positive rates presented in panel B. The third curve in that panel shows the rate of positive predictions, corresponding to the proportion of candidate patients that would be subjected to testing. Subsequent considerations will show that choosing a threshold of 0.5 (dotted vertical line) is a reasonable, although not necessarily optimal strategy for the problem at hand. The rate of positive predictions for this threshold is 0.38. Dividing the true-positive rate by the false-positive rate yields the likelihood ratio 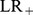, which is shown in panel C (only considering thresholds with more than 20 positive predictions). For a threshold of 0.5, this ratio is 2.11, which means that the odds of getting a positive test result are more than doubled.

The curves in Fig. 2 D show the positive predictive value (black) and the negative predictive value (gray). To ensure a certain minimum quality of the estimated values, only thresholds with more than 20 positive or negative predictions, respectively, were considered. The choice of a small threshold is tantamount to making a positive prediction for almost all patients. This explains why, for 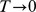, the positive predictive value) corresponds to the prevalence of a positive glycerol test, which is 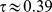 for the present data (lower dotted line). Starting from this level, the positive predictive value tends to increase with increasing threshold. The choice of a threshold close to one, by contrast, gives rise to a negative prediction for almost all patients. Thus, the negative predictive value for 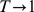 is 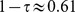 (upper dotted line).

**Figure 3:**
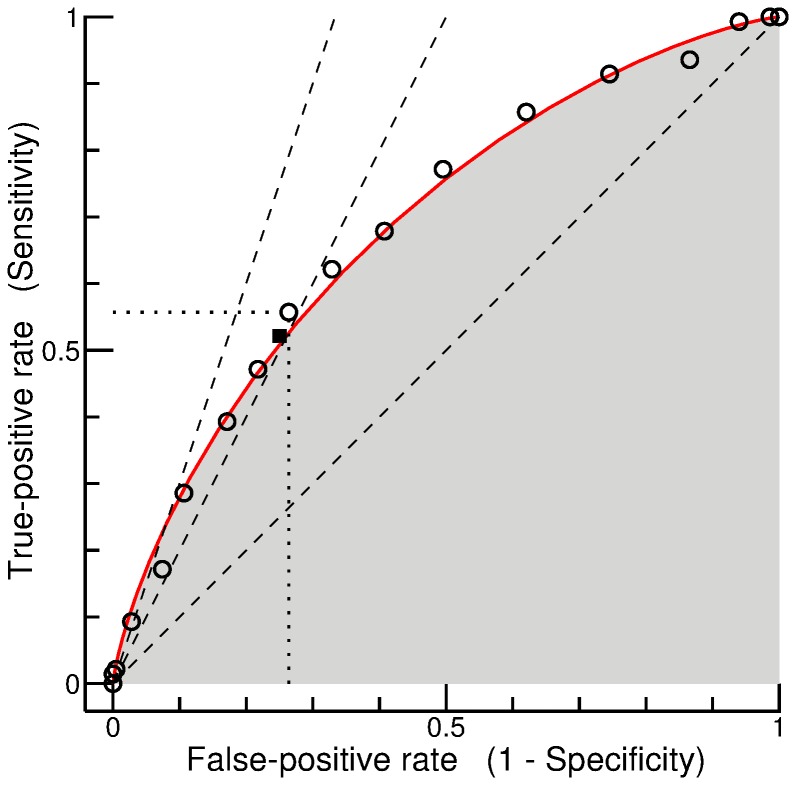
Receiver operating characteristic. The open circles represent the false-positive and true-positive rates displayed in Fig. 2 B, whereas the filled square represents the rule of thumb considered in Fig. 1. The three dashed lines indicate specific values of the likelihood ratio 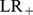 (1, 2, and 3).

A ROC-space representation of the the data points in Fig. 2 B is provided in Fig. 3 (circles). Dotted lines mark the data point corresponding to a threshold of 0.5. The smooth curve represents a simple Gaussian model with an index of detectability (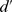) of 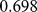 (the value was calculated from the estimated area under the curve, as desribed in the context of Eq. (14)). Dashed lines indicate different levels of the likelihood ratio 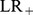, namely 1 (the diagonal), 2, and 3. It can be concluded that, for 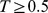, the likelihood ratio 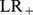 is typically between 2 and 3 (also shown in Fig. 2 C). The filled square represents the above-mentioned rule of thumb. Remarkably, this simple rule appears to perform similarly to the investigated K-nearest neighbor approach for a threshold of 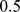.

The question remains how the investigated approach compares to alternative prediction methods. An answer can be obtained by comparing the AUC values compiled in Tab. 2. The first column gives a mnemonic for the predictor space, the other columns provide the AUC for the K-nearest neighbor approach and two variants of logistic regression: one working only with the original predictors (‘linear’), the other using the predictors squared as additional predictors (‘quadratic’). The mnemonics 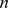
F (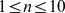) refer to investigations in which the hearing losses at the lowest 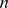 frequencies served as predictors. This means that 7F refers to the predictor space considered in Figs. 2 and 3. The mnemonic LH refers to a two-dimensional space with the mean low-frequency (L) and the mean high-frequency hearing loss (H) as predictors (considered in Fig. 8 of our previous article [14]). The space LH- is identical, except that the predictor H is replaced by the difference of H and L (considered in Fig. 1 of the present article). The predictor space 7F3d operates on the same frequency range as 7F, but the thresholds at neighboring frequencies were averaged (pairwise, except for the three lowest frequencies), which reduced the dimensionality from 7 to 3. The table suggests that, in spaces of low dimensionality, the performance of logistic regression can be moderately improved by including the squared predictors. All in all, however, the K-nearest neighbor approach is superior. Its performance improves as the number of frequencies increases, but including more than 6 frequencies appears to be detrimental, which would mean that the 7F space considered in Figs. 2 and 3 is suboptimal. A comparison of the spaces 7F and 7F3d suggests that reducing the dimensionality of the predictor space is advantageous mainly for the quadratic version of logistic regression.

**Table 2:** Comparison of various prediction methods in terms of the area under the ROC curve (AUC).

space	K-nearest	logistic regression	
	neighbor	linear	quadratic
1F	0.594	0.600	0.603
2F	0.624	0.609	0.629
3F	0.688	0.615	0.628
4F	0.667	0.616	0.623
5F	0.688	0.615	0.621
6F	0.704	0.641	0.635
7F	0.689	0.636	0.628
8F	0.678	0.631	0.619
9F	0.652	0.629	0.615
10F	0.655	0.623	0.605
LH	0.634	0.635	0.648
LH-	0.657	0.635	0.650
7F3d	0.690	0.655	0.674

### Cost-benefit Analyses

**Figure 4:**
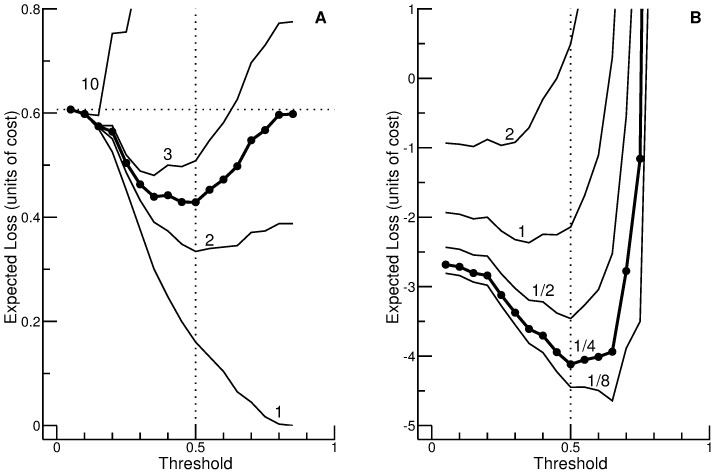
Expected loss as a function of threshold (cost-benefit analysis for the glycerol test). (A) Analysis based on the loss matrix defined in Eq. (18). The cost for the test is 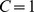, a negative test result has no benefit (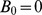), and a positive test result has the benefit indicated by the number attached to each of the four standard curves (1, 2, 3, and 10). In the case of the highlighted curve (thick with filled symbols), the benefit is the reciprocal of the prevalence of a positive test result. (B) Analysis based on the loss matrix defined in Eq. (21). The cost for performing the test is 1 and the benefit of a positive test result is 10. The numbers attached to the curves indicate the cost for screening.

In what follows, it is investigated how the optimal threshold for performing the glycerol test depends on the assumptions about cost and benefit. The curves in Fig. 4 A, showing the expected loss as a function of threshold, were calculated for the loss matrix defined in Eq. (18), under the assumption that the cost for the test is 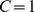 and that a negative outcome has no benefit, i.e., 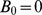. The numbers attached to the curves (1, 2, 3, and 10) indicate the assumed benefit of a positive outcome, 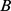. Since a benefit of 1 merely recoups the cost for testing, the expected loss increases as the threshold decreases, owing to the increasing proportion of patients for whom no benefit could be achieved. The optimal strategy, therefore, is to refrain from testing. For a benefit of 2, a flat minimum can be observed at a threshold of 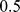. The highlighted curve (thick with filled symbols) represents the special case that the benefit is equal to the reciprocal of the prevalence of a positive test result, i.e., 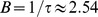. The expected loss for 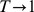 is obtained by multiplying 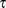 (now corresponding to the false negative-rate) by 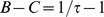, yielding 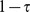. This is also the expected loss for 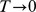 (irrespective of the assumed benefit). The minimal loss is obtained for a threshold of 0.5 or somewhat below. For a benefit of 3, the optimal threshold is about 0.35, and refraining from testing (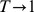) is worse than testing all subjects (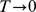). For a benefit of 10, the optimal strategy would be to test almost every patient, because the missed benefit from a false-negative decision represents a quite significant loss.

Figure 4B refers to the situation that a scientific study with a fixed number of subjects would be most beneficial if an embedded glycerol test has a positive outcome in as many subjects as possible (see Eq. (21)). The cost for performing the test is, again, assumed to be 1, whereas the benefit of a positive test result is set to 10. The numbers attached to the curves (1/8, 1/4, 1/2, 1, and 2) indicate the cost for screening, 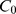. In the case of the highlighted curve (thick with filled symbols), which may serve as a reference, the cost for screening is significantly smaller than the cost for actually doing the test, but it is not negligible (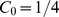). A threshold of about 0.5 turns out to be optimal in this situation. Doubling the cost for screening does not fundamentally change the situation, whereas halving shifts the optimal threshold to the right, meaning that the choice of subjects should become more selective. As to be expected, screening for suitable subjects is of limited value if this causes the same cost as actually doing the test (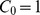), and screening becomes counterproductive if the cost is higher (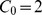).

## Discussion

### Decision Making based on the Cost-benefit Ratio

The predictive modeling approaches considered in this article use the experiences with previous patients for estimating the probability that performing a specific diagnostic test in a new patient will have a positive outcome. If the model underlying the prediction were perfect, the optimal strategy would be to test a patient whenever the estimated probability exceeds the threshold defined in Eq. (6), which corresponds to the cost-benefit ratio 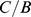 (under the simplifying assumption that a negative test result has no benefit). However, if the goodness of the predictive model is unknown, this simple rule is suitable only for qualitative considerations. Problems are to be expected, for example, when a K-nearest neighbor prediction is to be made for a point located in the periphery of the points representing the previous patients: The estimated probability may then be representative for the center of the 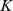 neighbors, but not for the eccentrically placed point of interest. A cost-benefit analysis using cross validation (as in Fig. 4) does not only assess the *true* performance of the model, but also gives an idea of how suboptimal decisions deteriorate the performance.

Instead of considering the expected utility of the diagnostic test, we modeled its negative counterpart: the expected loss. It is, of course, not possible to unconditionally generalize the results obtained for the illustrative example presented above, the glycerol test. However, some conclusions are probably valid for other diagnostic tests as well, especially since the parameters in the loss matrix are, as a rule, not precisely known. Thus, a more qualitative interpretation of our numerical results is advisable anyway. Regarding the analysis presented in Fig. 4 A it appears appropriate to distinguish three major conditions. Firstly, the benefit of a positive test result may very clearly exceed the cost (e.g., 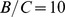). Excluding a patient from testing because of an erroneously predicted negative outcome would cause a significant loss, and so a low threshold has to be chosen (e.g., 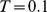, in accordance with Eq. (6)), which means that almost every patient is tested. Secondly, the benefit may not be significantly higher than the cost. In that case, it is advisable to choose a threshold close to 1 (again in accordance with Eq. (6)), which corresponds to refraining from testing. Thirdly, the benefit may be in between the two extremes (e.g., benefit-cost ratio of 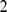 or 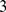). A threshold of 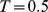 may then be considered as a reasonable default choice. Depending on the cost-benefit ratio, this choice may be suboptimal, but the disadvantage compared to the optimal choice can be expected to be small. A particularly interesting situation arises if the benefit-cost ratio equals the reciprocal of the prevalence of a positive test result (thick curve with filled circles in Fig. 4 A): Testing all patients (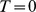) and testing no patient (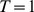) causes exactly the same loss, which is greater than the loss for any intermediate threshold. With respect to decision making this means that testing a subpopulation selected by predictive modeling is, in any case, better than the two extreme alternatives: testing each patient or no patient at all.

### Comparison with Related Work

Our approach is closely related to decision curve analysis as proposed by Vickers and Elkin [41]. While we use a loss matrix, they assign a ‘value’ to each outcome of a simple decision tree, which is, of course, equivalent. Moreover, their equation (1) corresponds to our equation (4). Remarkably, the unnumbered equation on page 567 of their article is a special case of our equation (17). To show this, we transcribe their equation using our own notation (we also apply their Eq. (1) and account for the fact they fixed 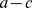 at 1):
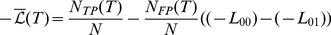
(22)


The equation can be rewritten as
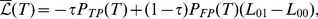
(23)which is equivalent to (17) if 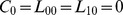 and 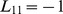. Note that the only free parameter in the loss matrix, 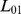, can be interpreted as a cost-benefit ratio. The assumption 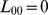 (also made in the present article) is without any reasonable alternative. Worded in neutral terms it means that refraining from action in cases where no action is required causes a loss of zero. The assumption 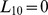 in the approach by Vickers and Elkin is consistent with our definition of a loss matrix in Eq. (20). Worded in neutral terms again, the zero of the loss scale is assigned to the situation that, regardless of any predictive information, no action is taken. By contrast, in the loss matrix defined in Eq. (18) we assume 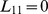, which means that the zero is assigned to the hypothetical situation that decisions about an envisaged action are made using a perfect classifier. However, as already explained in the context of Eq. (17), the definition of the zero point is irrelevant when comparing two methods. In conclusion, up to here our approach is basically equivalent to that of Vickers and Elkin [41].

The crucial difference is the way Eq. (4) is handled. Vickers and Elkin [41] assign a pivotal role to this equation in that they consider cost-benefit ratio and threshold probability as measures that can be converted into each other as desired. By contrast, we consider Eq. (4) as a theoretical relationship that cannot be expected to be strictly valid when dealing with real-world problems. We therefore ignore the equation in the first instance and consider threshold probability and benefit-cost ratio as parameters that can be independently varied. Plotting a family of curves as in Fig. 4 A is a convenient means to visualize how these two parameters affect the expected loss. Under ideal conditions, each curve would show a minimum at the threshold predicted by Eq. (4). Thus, the minima of the curves would be located on a graph comparable to the decision curve of Vickers and Elkin (of course, the fact remains that we consider loss rather than ‘value’ and that we define the zero point in a different way). Although our results roughly confirm this expectation, two aspects prevent perfect agreement. First, the optimal threshold calculated using Eq. (4) refers to the expected value of the loss, whereas our estimations are based on limited data. Second, the benefit-cost ratio is a continuous variable, whereas, for the K-nearest neighbor method considered in Fig. 4 A, the threshold probability is of a discrete nature.

Despite these formal discrepancies, it can be assumed that our approach and that of Vickers and Elkin [41] generally lead to similar conclusions. Thus, from the users’ point of view the differences between the two approaches are chiefly of a practical nature. In the approach by Vickers and Elkin, the user is assumed to already have an opinion about the threshold probability (and thus, implicitly, also about the cost-benefit ratio). Decision curve analysis can then be used to check whether it is sensible to apply this threshold to decision making using a specific predictive model. By contrast, our approach allows for the possibility that a user might have nothing more than a vague idea of the benefit-cost ratio. The questions asked under such circumstances are inevitably less focused, and a multi-step procedure might be appropriate. In the first step, it could be investigated for which range of benefit-cost ratios the predictive model promises to be beneficial. Supposing that this range includes the presumed benefit-cost ratio, the next step would be to determine the optimal threshold. Finally, it might be of interest to ask whether this threshold would be acceptable also for slightly different assumptions about the benefit-cost ratio. A plot such as the one in Fig. 4 A appears to be well-suited for considerations of this kind.

We are not aware of other articles coming as close to ours as does that of Vickers and Elkin [41]. But, of course, many authors considered related problems. For example, Moskowitz and Pepe [42] proposed a method for quantifying and comparing predictive accuracy of continuous prognostic factors. Their work differs from ours in that they consider single factors, whereas we consider multiple factors. Nevertheless, their idea of a standardized scale based on the cumulative distribution function of the respective prognostic factor could be useful also in the context of our K-nearest neighbor approach, because that way it would be possible to build up meaningful predictor spaces from factors of quite different nature (such as hearing loss and age). Another difference between our approach and that of Moskowitz and Pepe [42] is that we pursue loss minimization, whereas they advocate considering positive and negative predictive values. For the sake of conciseness, we abstain from comprehensively reviewing possible alternatives to our methodological approach. Instead, we refer to a review article by Gerds et al. [43]. They not only illuminate the broader context, but also present a unifying framework that shows clear parallels to our approach. Risk prediction models, as considered in their article, use prognostic factors available at time 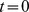 to determine the probability of a patient having a certain status at time 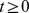. In our article, ‘status’ refers to the outcome of the glycerol test, and the prediction is evidently made for time 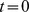. Amongst other things, Gerds et al. [43] compare traditional methods such as logistic or Cox regression with machine learning concepts such as classification trees and random forests. The former belong to the culture of data modeling, whereas the latter, just as the nearest neighbor method considered in the present article, belong to the culture of algorithmic modeling [44]. Gerds et al. [43] urge that performance measures should be applicable to all statistical cultures, and they regard crossvalidation and bootstrap to be the heart of internal validation. Our study is in line with these concepts. Moreover, their idea that the predicted probabilities can be considered as a new ‘single continuous marker’ corresponds to our interpretation of predictive modeling as a virtual screening test.

### Comparison of Predictive Modeling Approaches

Diagnostic testing is most useful when the presence of disease is neither very likely nor very unlikely [1]. The information theoretic explanation of this fact is that a test with a binary result (Yes/No decision) is most informative if the two alternatives have the same probability, because this is the condition under which the binary entropy function reaches its maximum (corresponding to one bit) [45]. The glycerol test considered here approximately satisfies this condition: The prevalence of a positive test result was almost 0.4 for the population of candidate patients, and with predictive modeling the positive predictive value increased up to about 0.6 (see Fig. 2 D), which means that the odds of obtaining a positive test result were more than doubled. It should be noted that this improvement was achieved with a relative simple predictive model, which was defined *before* the data were analyzed. Table 2 suggests that this model (K-nearest neighbor approach for the space 7F) is probably suboptimal, which means that a better performance might be attainable by employing a different combination of predictors, possibly with optimized weights (although the gain to be made by weighting may not be worth the extra effort [46]). However, in view of the limited amount of data available, we refrained from systematically seeking for an optimal model, because this would involve the risk of overfitting, with the consequence that the optimized model may not generalize well to new data [30]. Besides, as long as the analysis depends on rough guesses of cost and benefit, reaching indisputable conclusions is impossible anyway.

But these limitations do not preclude a more general comparison of methods. A convenient possibility is to calculate the area under the ROC curve (AUC), although the approach must be considered with reservation because this widely used measure was shown to be incoherent [47], [48]. We start the discussion of Tab. 2 by considering the predictor space LH-. The visualization of this space in Fig. 1 shows that the probability of a positive test result is clearly enhanced if the predictor represented by the horizontal axis (low-frequency hearing loss) has an intermediate value. A linear regression model is not able to adequately account for this observation, and therefore it is not surprising that including the predictors squared as additional predictors yields an improvement: Even though the effect is relatively small, the quadratic version of logistic regression approximately reaches the performance of the K-nearest neighbor approach in this space. The latter method is distinguished by the fact that predictions are made locally, on the basis of small subsets of data. This gives the method a high flexibility, which may explain why it turned out to be superior in spaces of higher dimensionality (greater than two). An additional reason for this superiority is that the inclusion of quadratic terms in the logistic regression approach is apparently counterproductive when the dimension of the space is greater than five, presumably because generalization to new data becomes a problem.

A comparison of the spaces 1F to 10F confirms the initial hypothesis that the hearing thresholds at the three highest frequencies have no predictive value. To be exact, Tab. 2 suggests that only the thresholds at the six lowest frequencies contribute substantial information (K-nearest neighbor approach and linear logistic regression are consistent in this respect). A comparison of the spaces 7F and 7F3d suggests that averaging over appropriately chosen frequency bands can improve the performances of the two variants of logistic regression, whereas there is no obvious advantage for the K-nearest neighbor approach. However, for other data transformations the situation may be the other way around, as suggested by a comparison of the spaces LH and LH-. The latter was derived from the former by subtracting the two predictors and substituting the result for one of the original predictors. While this step had basically no effect on the performances of the two logistic regression approaches, the performance of the K-nearest neighbor approach slightly improved. Thus, it is conceivable that more complex and possibly nonlinear transformations of the data (resulting in predictors that describe the shape of the pretest audiogram in terms of, e.g., ‘curvature’ or ‘frequency of the steepest gradient’) would allow us to outperform the approaches considered in Tab. 2. Nevertheless, it appears doubtful that striking improvements are possible without introducing additional predictors. To better characterize the pretest hearing status, it might be helpful to consider, for example, measures of frequency selectivity and compression [49].

### Predictive Modeling versus Simple Rule of Thumb

A simple rule of thumb turned out to be competitive with the much more sophisticated predictive modeling approaches. This result was not expected, but it is not too surprising either. There are probably two reasons that contributed to this outcome. First, the use of expert knowledge of the condition tested for avoids the need to make potentially unreliable estimations based on a limited amount of data (e.g., considering the 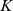 nearest neighbors). If the key information contained in the predictors can be captured by a simple rule (which, in the present example, apparently concerns the relationship between low- and high-frequency hearing loss), it may be difficult for unspecific prediction methods to achieve a significantly better performance. The argument is supported by the fact that the predictor space underlying the rule of thumb (LH-) is only a mediocre choice (see K-nearest neighbor method in Tab. 2). In reverse, this means that it might be worthwhile to seek for a rule of thumb operating in a different low-dimensional space (such as 7F3d). Second, the data used for establishing and evaluating the rule of thumb were the same. Thus, it might be the case that applying the rule to new data would result in a somewhat lower performance. The predictive modeling approaches, by contrast, were evaluated using cross-validation so that there is no reason to assume that the performance will differ for a population of new patients, provided that the external conditions and the inclusion criteria remain the same. Regardless of these considerations, predictive modeling has the principle advantage that the proportion of patients tested can easily be varied by adjusting the threshold, thus allowing us to account for the presumed cost-benefit ratio of the test. The latter may critically differ even from patient to patient. For example, a test providing mainly prognostic information [50] is beneficial only to patients who want to know the prognosis.

## Conclusions

The performance of a diagnostic test is commonly characterized in terms of its sensitivity and specificity. However, these two well-known measures are not necessarily sufficient for assessing how useful a test is in daily practice. To reach a well-founded conclusion, it is indispensable to consider additional factors such as the cost for performing the test and the benefit of a positive and a negative test result, respectively. Of crucial importance is also the prevalence of the condition tested for, which, by definition, depends on the population of patients. Thus, the utility of a test may crucially depend on how the tested patients are selected. As yet, the selection is typically based on relatively coarse criteria. The patients may, for example, be suspected of having a certain disease. Predictive modeling opens up the possibility to come to individualized decisions, with the consequence that a diagnostic test may be considered useful for a particular patient even when testing a broader population of patients with the same suspected diagnosis is discussed controversially. Moreover, if alternative tests are available, predictive modeling may help to choose the one that is most promising for the particular patient.

## Supporting Information

File S1 **Zipped folder containing Matlab scripts that may be used to generate simplified versions of the Figures and of** **Table 2** **.** The file provides the data and the Matlab code.
(ZIP)
