# Correction: Predictive Modeling for Diagnostic Tests with High Specificity, but Low Sensitivity: A Study of the Glycerol Test in Patients with Suspected Menière’s Disease

**DOI:** 10.1371/annotation/430a6ff5-9e80-4767-b423-1b7de0d484cc

**Published:** 2014-01-06

**Authors:** Bernd Lütkenhöner, Türker Basel

An error occurred in the legend of Figure 1 during the preparation of this article for publication. A reference is missing from the sentence beginning "The area framed by thick gray lines with downward arrows represents a simple rule of thumb[?]...". The sentence should instead read:

"The area framed by thick gray lines with downward arrows represents a simple rule of thumb [14] according to which a glycerol test should be considered as a diagnostic option when a patient with suspected Menière’s disease has a mean low-frequency hearing loss between 30 and 70 dB and a mean high-frequency hearing loss that does not exceed the mean low-frequency loss.

